# Socio-Economic Inequalities of Childhood Stunting in Rwanda: Time Trend Analysis of Demographic and Health Surveys, 2000 to 2019

**DOI:** 10.1007/s44197-026-00566-3

**Published:** 2026-04-29

**Authors:** Albert Ndagijimana, Leonardo Z. Ferreira, Torbjörn Lind, Aluisio J. D. Barros

**Affiliations:** 1 Department of Clinical Sciences, Umeå University, Paediatrics, Umeå, Sweden; 2 College of Medicine and Health Sciences, School of Public Health, University of Rwanda, Kigali, Rwanda; 3 International Center for Equity in Health, Universidade Federal de Pelotas, Pelotas, Brazil

**Keywords:** Stunting, Childhood, Nutrition, Inequality, Rwanda, Socioeconomic

## Abstract

**Background:**

Childhood stunting, defined as a height-for-age Z-score below − 2 standard deviations, remains a major public health issue in Rwanda, with one in three children under-five affected. Although national prevalence of stunting has declined over the past two decades, less is known about how trends compare across population groups. This study assessed trends in childhood stunting inequalities in Rwanda from 2000 to 2019 using absolute and relative inequality measures across five equity dimensions (wealth, women’s education, place of residence, sex, and regions or provinces).

**Methods:**

We analyzed data from five rounds of the Rwanda Demographic and Health Surveys (2000–2019). Stunting prevalence was disaggregated by wealth quintiles, women’s education, place of residence, child’s gender, and region/province. Absolute and relative inequality measures included the slope index of inequality (SII), concentration index (CIX), absolute difference, and the weighted mean difference to the mean (WMDM). Population attributable risk (PAR) and population attributable fraction (PAF) were estimated to assess public health impact.

**Results:**

Stunting prevalence in Rwanda decreased by 31.0% from 2000 (47.9%) to 2019 (33.1%). However, inequalities widened, with faster progress among the wealthiest, reaching their peak in 2019 (SII = -41.7, CIX = -21.9). Women’s education inequalities persisted in favor of the highly educated (SII = − 29.7 and CIX worsening to -9.2 in 2019), urban areas (16-point absolute difference), and boys (7.8-point absolute difference). Regional disparities were relatively stable, with WMDM around 5.0% points, Kigali having the lowest rate. The measures of impact show wealth had the strongest effect, with a PAR of 23.2% points and PAF of 69.0%; respectively reflecting the amount of stunting rates that are attributable to being in the poorest categories and what could be eliminated if all children were in the wealthiest categories.

**Conclusion:**

Despite overall national progress in reducing stunting, social and economic inequalities remained apparent and widened between 2000 and 2019. Children from the poorest households and those with less educated mothers remain disproportionately affected. To achieve the global nutrition target by 2030, Rwanda must strengthen equity-focused interventions to reach the most vulnerable groups.

**Supplementary Information:**

The online version contains supplementary material available at 10.1007/s44197-026-00566-3.

## Background

Childhood stunting, defined as a height-for-age Z-score below − 2 standard deviations, remains a public health challenge worldwide, particularly in low- and middle-income countries. Globally, 150.2 million children under five years of age are stunted, with the highest prevalence observed in sub-Saharan Africa and South Asia [1]. Stunting has irreversible long-term consequences on physical and cognitive development, educational attainment, and economic productivity [2–4]. In Rwanda, the most recent demographic and health survey (DHS) indicates that one in every five children under the age of five years is stunted [5].

Globally, childhood stunting arises from several factors at individual, household, and structural levels. Recent global syntheses emphasise inadequate dietary intake, maternal undernutrition, recurrent infections, poor water, sanitation and hygiene conditions, limited access to health services, and low socioeconomic status [6, 7]. Large comparative analyses and systematic reviews consistently show that stunting is socially patterned, with substantially higher prevalence among children living in poor households, in rural areas, and those born to mothers with low educational attainment [6, 8, 9]. Sex-based differences are also widely reported, with boys experiencing higher stunting prevalence than girls across most low- and middle-income settings, a pattern attributed to a combination of biological vulnerability and heightened sensitivity to adverse early-life environments [8, 10].

In Rwanda, recent empirical studies echo these global patterns. Analyses based on national survey data and systematic reviews report higher stunting prevalence among children living in poor households, those born to women with lower levels of education, children residing in rural areas, and male children, alongside persistent differences across provinces [11–14]. Despite growing descriptive evidence, no studies have applied rigorous social epidemiological approaches to quantify socioeconomic inequalities in childhood stunting at the country-wide level using summary measures of inequality that allow comparison over time. There is evidence of higher stunting rates in under-fives from poor households, with low women’s education, in rural settings, and among male children with older age [11, 13, 15–17], as well as in some provinces [16, 17]. Therefore, in the context of a country like Rwanda, with a relatively slow decline in stunting prevalence compared with the global target of reducing stunting prevalence by 40% by 2030 [18], it is important to understand how this public health problem is distributed across key social and economic dimensions, for guiding equity-oriented policy and programming.

Against this background, we assessed the magnitude of absolute and relative socioeconomic inequalities in stunting and whether progress has been made in reducing such inequalities using nationally representative surveys, to guide public health actions and accelerate the efforts towards the 2023 target [18].

## Methods

### Study Area

Rwanda is a small, landlocked country located in East Africa. It is divided into five provinces and 30 districts. The 2022 Rwanda Population and Housing Census shows a population of 13,246,394 people, with high density (503 inhabitants per square kilometer as of August 2022) and an average annual growth rate of 2.3% between 2012 and 2022 [19]. Approximately a third of residents (27.9%) live in urban areas. The City of Kigali is the most urbanized Province (86.9%), while the Southern Province has the smallest proportion of urban population (14.8%) [19]. Almost 13% of the population is under the age of five. Overall, 49% of the women aged 15–49 years have an education less than primary level, 18% have a primary level, 21% have some secondary level, 7% have completed secondary, and 4% have more than a secondary level [17]. More than half of the employment relies on agricultural activities [17]. More specifically, 69.2% of the population in rural areas is in the first three lower wealth quintiles, as compared with 14.9% of the population in urban areas [17].

### Data

We used data from five Rwanda demographic and health surveys (RDHS) carried out in 2000, 2005, 2010, 2015, and 2019. The survey targeted women aged 15–49, men aged 15–59, and children under five years. Data were collected on fertility levels and preferences; contraceptive use; maternal and child health; infant, child, and neonatal mortality levels; maternal mortality; gender; nutrition; awareness about HIV/AIDS; self-reported sexually transmitted infections (STIs); and other health issues relevant to the achievement of the Sustainable Development Goals. The surveys use a two-stage sampling strategy: first, clusters (enumeration areas) were randomly selected, and then households were selected systematically within those clusters to ensure representativeness. Further details about the surveys and their sampling frame are presented in each survey report [20, 21].

### Outcome and Equity Dimensions

We estimated the prevalence of stunting for all under-five children who slept in the household the night before the survey. Stunting is defined as a height-for-age Z-score less than − 2 standard deviations, according to the 2006 World Health Organization Child Growth Standards [22] and the DHS Statistics guide [23], with children categorized as stunted or not stunted.

We disaggregated childhood stunting by five equity dimensions: economic status (wealth quintiles), women’s education, place of residence, province, and sex of the child [24, 25]. The household economic status was classified into five ordered quintiles, from the poorest 20% (first quintile) to the wealthiest (fifth quintile), using an asset-based wealth score, which is calculated using principal component analysis based on household ownership of assets, housing characteristics, and access to services. Women’s education was categorized into three subgroups (no education, primary education, and secondary or higher). The place of residence was classified as rural or urban, according to the country’s definition. The child’s sex was coded as male or female, according to the mother’s report. The provinces were nominally recorded as East, Kigali City, North, South, and West.

### Statistical Analysis

Descriptive statistics with frequencies and percentages were used to portray trends in the prevalence of childhood stunting by different equity dimensions in Rwanda between 2000 and 2019.

As simple measures of inequalities in stunting, difference (D) and ratio (R) were used. Unweighted, these measures were respectively used to show absolute and relative inequalities for all equity dimensions, comparing the extreme subgroups. For any health indicator, absolute inequality measures reflect the magnitude of the difference in health outcomes between subgroups. Conversely, relative inequality measures provide a proportional comparison of health outcomes across different subgroups [26]. Then, the slope index of inequality (SII) was used as a complex measure of absolute inequality, and the concentration index (CIX) as a complex measure of relative inequality. SII and the CIX were calculated for the two ordered socioeconomic dimensions, wealth quintiles, and women’s education. SII measured the absolute difference in stunting prevalence between the wealthiest and poorest households. Negative values of the SII indicated higher prevalence among the poorest, while positive values indicated the opposite. If there is no inequality, SII is zero. The CIX indicated the relative distribution of stunting across socioeconomic groups, with negative values showing a concentration among the poor.

To assess the impact of public health inequality, population attributable risk (PAR) was estimated as an absolute measure, and population attributable fraction (PAF) was estimated as a relative measure. PAR was used to quantify the improvement in childhood stunting in a given setting that could be achieved if all population subgroups had the same rates as the reference subgroup (for instance, if the first four wealth quintiles had the same stunting prevalence as the fifth quintile, the wealthiest). On the other hand, the PAF was computed to indicate the proportion of stunting that could be improved under the same better conditions. Note that this interpretation assumes unicausality.

Subnational inequalities were estimated as absolute measures, using the weighted mean difference to the mean *(*WMDM). This is calculated as the average of absolute differences between each province’s prevalence and the national average, weighted by the province’s population size. Larger values indicate higher levels of inequality. A detailed explanation of these measures is presented elsewhere [26–28].

### Ethical Considerations

This study used data from the last RDHS (2000–2019). The RDHS protocols were reviewed and approved by the Rwanda National Ethics Committee and the ICF Institutional Review Board. All ethical principles were complied with in these surveys. Therefore, this study did not involve any direct interaction with human subjects.

## Results

### The Trend in Childhood Stunting Prevalence by Different Inequality Dimensions in Rwanda

Table 1 details the stunting prevalence over time and across five dimensions of inequality in children under five in Rwanda. The national stunting prevalence in children under five has significantly decreased by 31.0% from 2000 (47.9%, 95% CI 46.4%, 49.7%) to 2019 (33.1%, 95% CI 31.3%, 35.0%). Childhood stunting prevalence showed a clear socioeconomic pattern, decreasing monotonically with increasing wealth and women’s education. The prevalence was consistently higher in rural areas and among boys. Lower prevalence was consistently reported in Kigali City when compared to the other provinces. It is worth noting that the largest decreases in stunting were found in better-off subgroups, with a reduction of 68.1% among the wealthiest quintiles, compared to a mere 8.7% reduction in the poorest quintile. Similar patterns were observed for women’s education and urban/rural residence. Girls and the East province presented larger reductions compared to the other groups in each dimension.

**Table 1:** The trend in the prevalence of childhood stunting by different inequality dimensions in Rwanda, 2000–2019

Dimension of inequality	2000		2005		2010		2015		2019		% change (2000–2019)
	%	*N*	%	*N*	%	*N*	%	*N*	%	*N*	
National	48.0	6,758	51.5	3,940	44.3	4,369	38.3	3,816	33.1	4,050	31.0
Wealth quintile											
Poorest	53.1	2,242	61.1	767	54.1	952	49.2	947	48.5	965	8.7
Poorer	52.1	743	55.0	786	51.0	954	44.8	791	40.5	829	22.3
Middle	51.0	1,090	51.8	795	45.9	861	38.2	709	32.8	755	35.7
Wealthier	48.7	967	51.2	825	39.3	836	30.2	645	28.6	761	41.3
wealthiest	33.5	1,716	35.3	767	26.0	766	21.4	724	10.7	740	68.1
Woman’s education											
None	52.9	1,952	55.9	982	52.2	769	47.5	496	45.1	450	14.7
Primary	47.9	3,432	50.8	2,343	44.6	2,922	39.5	2,542	35.2	2,479	26.5
Secondary/higher	31.3	814	38.4	355	23.2	378	19.8	498	20.8	865	33.5
Place of residence											
Urban	33.3	1,512	38.4	798	27.3	598	24.3	814	19.8	841	40.5
Rural	50.7	5,246	53.6	3,142	46.6	3,771	40.9	3,002	35.8	3,209	29.4
Child’s sex											
Male	50.6	3,348	52.8	1,927	47.6	2,188	43.1	1,943	37.0	2,039	26.9
Female	45.5	3,410	50.2	2,013	41.1	2,181	33.4	1,873	29.2	2,011	35.8
Provinces											
Kigali City	-	-	-	-	23.3	472	23.4	435	21.3	454	8.6
South	-	-	-	-	42.3	1,111	41.6	981	32.7	930	22.7
West	-	-	-	-	50.0	1,045	45.0	931	40.2	1,017	19.6
North	-	-	-	-	50.7	689	39.3	531	40.5	649	20.1
East	-	-	-	-	44.2	1,052	35.0	938	28.8	1,000	34.8

*Notes*: %: stunting prevalence, N: unweighted sample size. Before 2005, Rwanda was subdivided into prefectures, not provinces. So, the comparison by province between 2000 and 2019 was not possible. For this dimension, the % change was calculated between 2010 and 2019

### Inequality in Childhood Stunting in Rwanda between 2000 and 2019

**Fig. 1:**
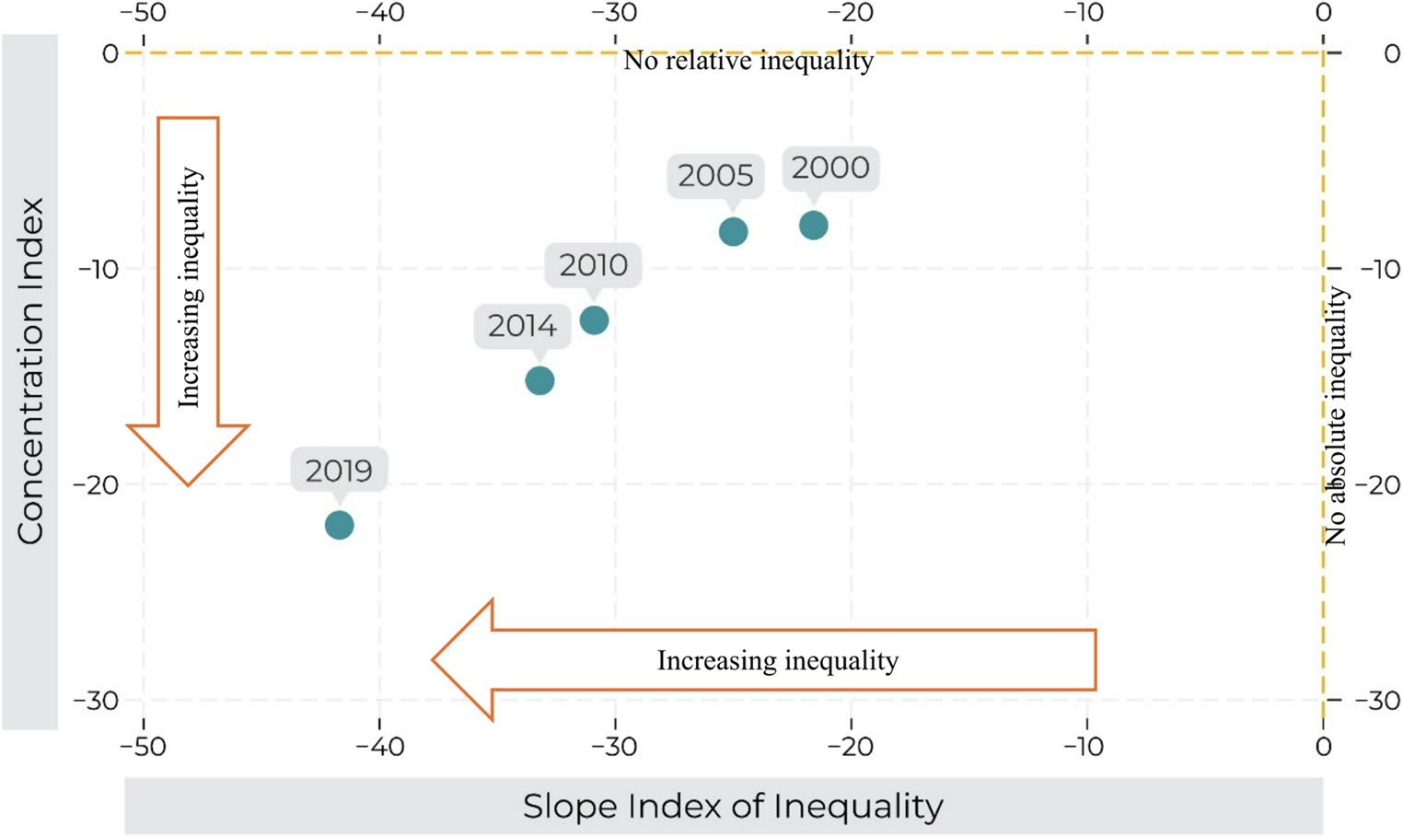
Absolute and relative measures of inequality in childhood stunting in Rwanda, by socioeconomic status (wealth quintiles), 2000–2019

Figure 1 plots the wealth-related SII and CIX for each time point. Both absolute and relative inequalities in child stunting have increased, reaching their peak in 2019 (SII = -41.7, CIX = -21.9) (Appendix 1).

**Fig. 2:**
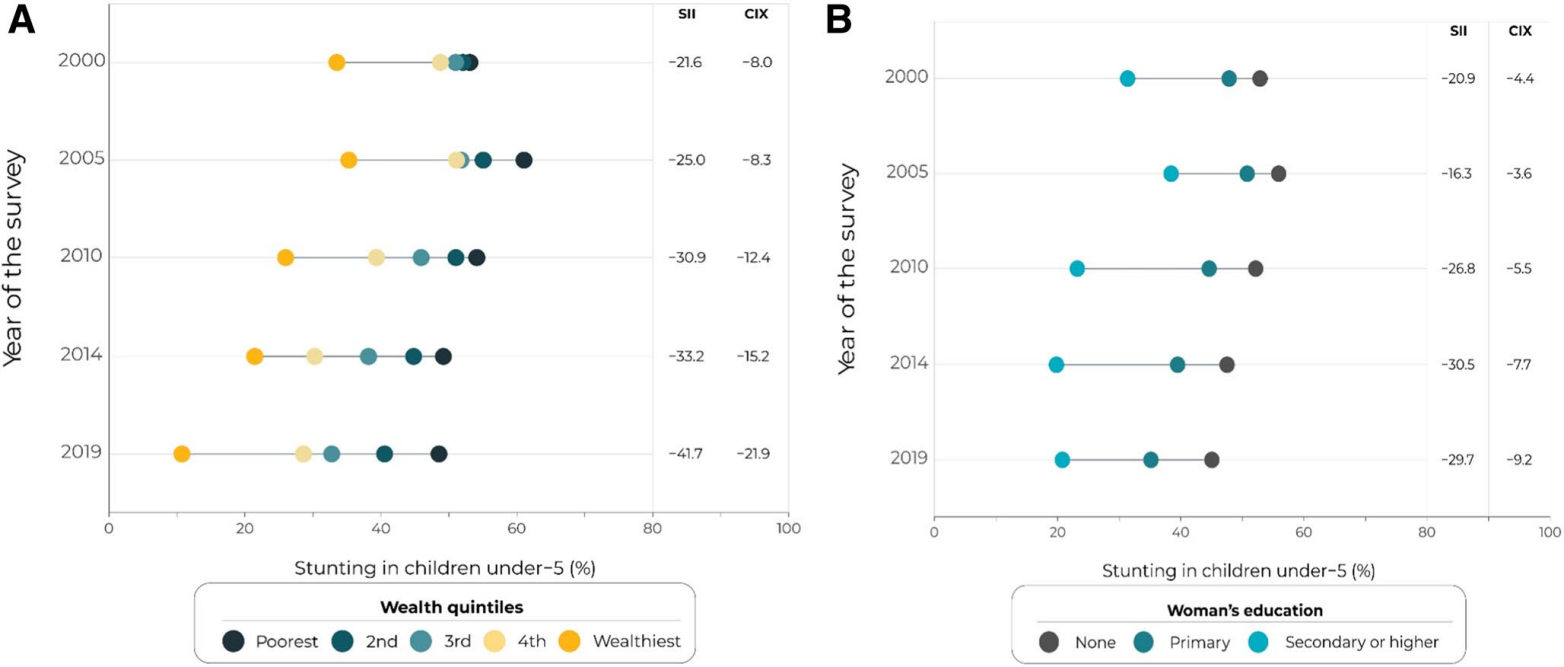
Equiplot of childhood stunting in Rwanda by wealth quintiles (**A**) and women’s education (**B**), with corresponding SII and CIX, 2000–2019

Figure 2 shows stunting prevalence for the five wealth quintiles (A) and women’s education (B) by survey year, along with SII and CIX estimates. The increase in absolute inequality is visualized as an increase in the distance between the dots from 2000 to 2019. The prevalence among the poorest barely changed over time, while there was an important reduction among the wealthiest. The middle groups, which started clumped together (a top inequality pattern), moved farther from each other, developing a pattern close to linear inequality. As for education, there was a gradual increase in absolute and relative inequalities, and persistent disparities between the three levels of women’s education. However, the change in pattern over time was less evident than with wealth.

**Fig. 3:**
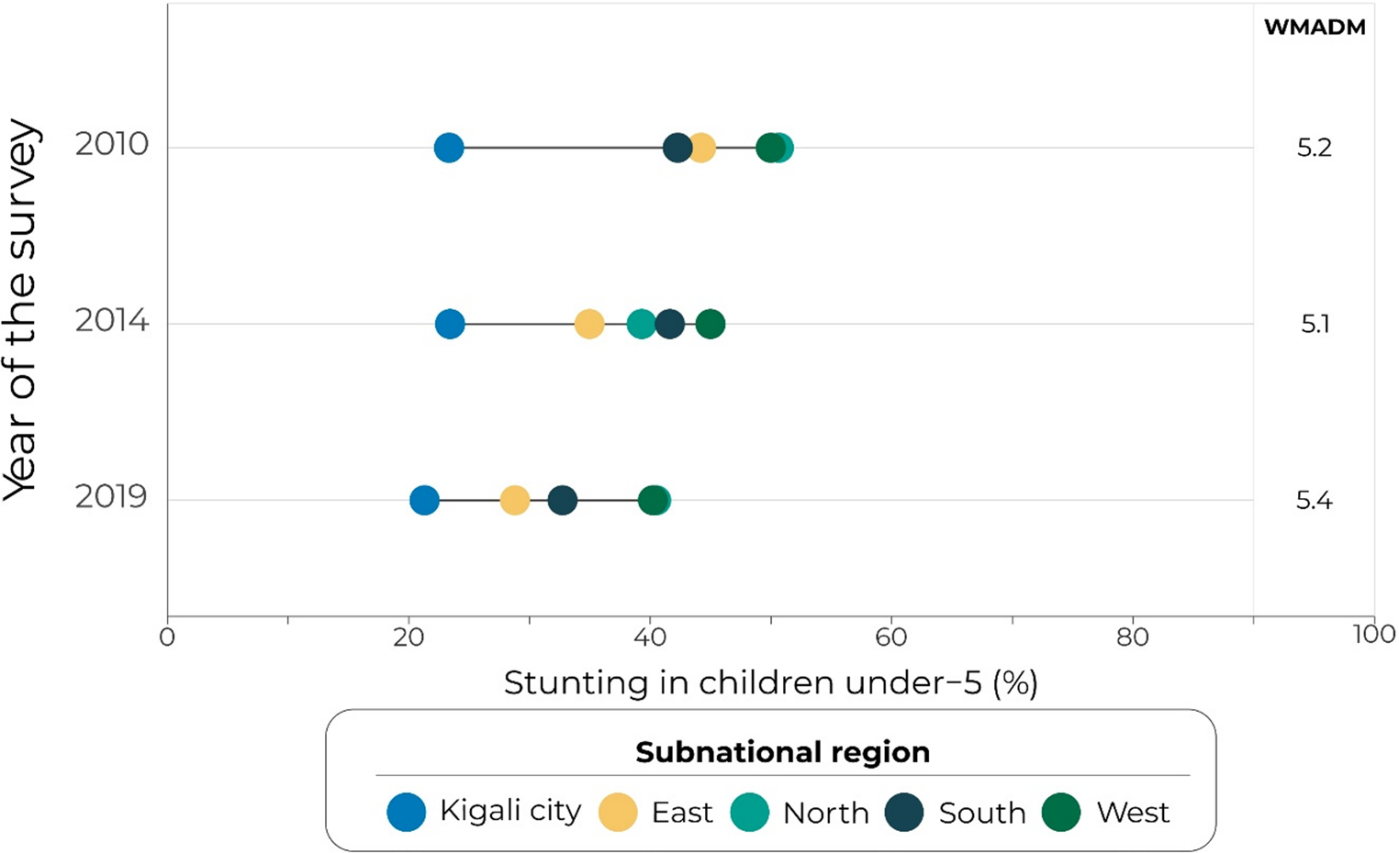
Equiplot for childhood stunting in Rwanda, by provinces, 2010–2019

Figure 3 shows regional disparities that were evident in 2010 between Kigali City, with the lowest prevalence, and other provinces. WMDM remained constant over time, with values of 5.3 in 2000 and 5.4 in 2019 (Appendix 1).

By place of residence, stunting prevalence remained consistently higher in rural areas, with disparities stable over time, as reflected by both difference and ratio (Appendix 1). When it comes to the child’s sex, boys consistently presented a higher prevalence than girls, with the difference increasing from 5.1% points in 2000 to 7.8 in 2019.

The PAF indicated wealth as the most impactful inequality measure for stunting in the country. Specifically, between 2000 and 2019, 32.1% and 69.0% of stunting cases could be attributed to the poorest category of households, 39% and 38% to having mothers with no education, 32.0% and 39.0% to living in rural areas, and 38.4% and 38.3% to living in provinces with the highest stunting prevalences. More specifically, the PAR values indicate that between 2000 and 2019, 14.9 to 23.2% points of stunting could be avoided/eliminated if all children were in the better-off category, 18.1 to 12.6% points if they had mothers with secondary or higher education, 15.0 to 13.0% points if they were all living in urban areas, and 18.0 to 13.0% points if they were all from Kigali City (consistent with the lowest prevalence over years) (Appendix 1).

## Discussion

This study examined trends in socioeconomic inequalities in childhood stunting in Rwanda between 2000 and 2019. Although overall stunting prevalence declined by 31% over the period, the reduction was uneven across population groups. The most substantial improvements occurred among children living in the wealthiest households and those whose mothers had higher levels of education, while children living in the poorest households experienced markedly slower progress. As a result, both absolute and relative inequalities in stunting widened over time, particularly along with wealth and educational gradients. Disparities by place of residence and child sex persisted, with consistently higher stunting prevalence observed among rural children and boys, while regional differences remained relatively stable, with Kigali City showing the lowest prevalence throughout. Together, these findings indicate that national progress in reducing childhood stunting has not been accompanied by proportional gains among the most disadvantaged groups, reinforcing the importance of examining equity alongside overall trends.

Rwanda continues to report one of the highest childhood stunting prevalence rates in the region, although levels are lower than in several other sub-Saharan African countries [1, 29]. While stunting declined over time, the average annual rate of reduction of about 1.7% remains well below the 4% trajectory required to meet the global target of reducing childhood stunting by 40% by 2030 [1], despite sustained economic growth and a marked decline in poverty, from 58.9% in 2000 to 27.4% in 2023 [30]. This divergence suggests that economic growth and poverty reduction have not translated proportionally into nutritional gains, highlighting the need for more explicitly pro-poor and equity-focused strategies to accelerate reductions in childhood stunting [31].

Our analysis showed that wealth-related inequalities in childhood stunting widened markedly between 2000 and 2019. Although national stunting prevalence declined, reductions were substantially greater among children in the wealthiest households, while those in the poorest households made much slower progress. Both absolute and relative measures indicated increasing pro-poor inequality over time. This pattern aligns with evidence from multiple low- and middle-income countries (LMICs), where declines in national stunting prevalence have often been accompanied by widening socioeconomic inequalities [6, 32, 33]. Similar trends have been reported in several sub-Saharan African countries, indicating that economic growth alone may disproportionately benefit better-off households unless equity-focused policies are in place [34]. In the Rwandan context, this finding suggests that broader socioeconomic improvements have not translated evenly into nutritional gains for the poorest households. Persistently high stunting prevalence among children living in poverty indicates that material deprivation, food insecurity, and constrained caregiving environments continue to shape child growth outcomes. Evidence is needed to assess the effectiveness of poverty alleviation programs such as the Vision 2020 Umurenge Program (VUP), established in 2008, which targets the most vulnerable families through cash transfers, public works employment, and financial services [33, 34], and the Girinka Program (“One cow per poor family”), established in 2006, which aims to increase household economic welfare and generate spillover effects in local communities [32], thereby addressing high stunting rates in poor households. Therefore, integrating nutrition-specific interventions with poverty alleviation, social protection, and livelihood-support programmes may help ensure that future reductions in stunting are more evenly distributed across socioeconomic groups [35, 36].

Large and persistent inequalities in childhood stunting were observed by women’s education. Despite overall declines in stunting prevalence between 2000 and 2019, children born to women with secondary or higher education experienced consistently lower prevalence and faster improvements than those born to women with no or only primary education. Recent global and regional evidence confirms maternal education as one of the most robust and consistent predictors of linear growth in early childhood. A 2024 systematic review and meta-analysis, including studies up to 2024, showed that higher maternal education was strongly associated with higher height-for-age z-scores and lower stunting risk in LMICs, with particularly strong effects in Africa and South Asia [37]. Similarly, recent DHS-based analyses from West Africa and South Asia continue to show persistent educational gradients in stunting despite national progress [38, 39]. It is commonly known that education is highly correlated with wealth, and it is reported as one of the main determinants of wealth inequalities in childhood stunting [40–42]. In Rwanda, the absolute and relative effects of education on stunting remain evident in the sense that educated mothers increase household income, and children are better-nourished [35, 36]. The persistence of educational inequalities suggests that gains in child nutrition are being transmitted intergenerationally, reinforcing disadvantage among children born to less-educated women. Therefore, long-term investment in girls’ education beyond primary level must be recognised as nutrition-sensitive interventions. In the shorter term, nutrition and caregiving support targeted at mothers with low educational attainment, delivered through community health workers and antenatal/postnatal services, may help mitigate intergenerational disadvantages.

Children living in rural areas consistently had higher stunting prevalence than those living in urban areas throughout the study period. Although stunting declined in both settings, the rural-urban gap showed little evidence of narrowing. Recent large multi-country analyses using DHS data from 2010 to 2024 confirm that rural residence remains strongly associated with higher odds of stunting across sub-Saharan Africa, even after adjustment for household wealth and maternal characteristics [9, 43]. A 2025 regional study further demonstrated that rural–urban disparities in stunting have been remarkably resilient over time, despite overall declines in prevalence across SSA [44]. In Rwanda, rural residence continues to capture layered disadvantages, including poverty, food insecurity, limited infrastructure, and constrained access to health and sanitation services. The persistence of rural-urban inequalities suggests that national improvements have not sufficiently addressed structural vulnerabilities affecting rural households. Therefore, closing rural-urban disparities will require explicitly rural-focused strategies, including strengthening nutrition-sensitive agriculture, improving access to water and sanitation, and enhancing the reach and capacity of community-based health and nutrition services in rural settings.

Across all survey rounds, boys consistently exhibited higher stunting prevalence than girls, with the absolute difference increasing over time. Recent global syntheses reaffirm that boys are more likely to be stunted than girls in most LMICs. A comprehensive narrative review and updated meta-analyses confirm higher biological susceptibility among boys, coupled with greater vulnerability to infections and environmental stressors in early life [8, 10]. In sub-Saharan Africa, the Great Lakes countries report the largest gender gaps with higher risks in boys, and more specifically in Rwanda and Burundi [45]. These sex differences remain evident in limited settings and have not diminished as national prevalence declines. In Rwanda, sex-based patterns of stunting are evident, with boys bearing the higher risk [15]. The persistence and widening of sex differences in Rwanda suggest that overall improvements in nutrition, caregiving, and living conditions have not been sufficient to offset male biological vulnerability. Rather than reflecting differential treatment, this pattern likely indicates that early-life environmental risks remain substantial. Therefore, rather than sex-specific targeting, interventions should prioritise improvements in early-life conditions, with an accent on the modifiable risk factors of gender gaps [45]. A focus on the first 1000 days of life remains relevant [46].

Regional disparities in childhood stunting were apparent but largely stable over time. Kigali City consistently exhibited the lowest prevalence, while other provinces maintained higher levels with limited convergence. Similarly, country regional disparities of childhood stunting are reported in different LMICs, reflecting entrenched structural differences rather than short-term variation [35, 47]. In Rwanda, the sustained advantage of Kigali City likely reflects concentrated development, relatively well-paying employment opportunities, better infrastructure, and improved access to services. The lack of convergence across other provinces suggests that spatial inequalities remain deeply embedded and resistant to uniform national approaches. Reducing regional disparities requires place-sensitive planning, with nutrition and social interventions adapted to provincial contexts. Prioritising high-burden provinces for intensified multisectoral investment may be necessary to accelerate equitable progress. Furthermore, within provinces, hidden disparities should also be targeted for focused interventions, after realizing that stunting is clustered [48].

This study’s strengths relate to the data sources, five reliable and nationally representative studies (DHS) that use robust, complex and consistent sampling strategies, ensuring our findings’ external validity. This is the first study to use both absolute and relative inequality measures to display inequalities across five dimensions in Rwanda, as per the current literature. Providing a complete snapshot of inequalities from different perspectives in childhood stunting in Rwanda can serve as a basis for targeted interventions. As limitations, while the DHS design allows for the identification of trends and associations, it does not permit causal inference or evaluation of the effectiveness of specific policies or interventions. In addition, although the five equity dimensions examined capture key social stratifiers commonly used in inequality monitoring, they do not encompass all determinants of childhood stunting reported in the literature, such as dietary intake, exposure to infectious diseases, maternal nutritional status, household food security, and caregiving practices. As a result, the observed inequalities likely reflect the combined influence of both measured and unmeasured factors. Also, we did not explore why stunting inequalities remain apparent in the country. Therefore, further studies should explore this aspect through different approaches, such as decomposition analyses, to determine factors/contributors to the inequality at the national level, and compare with what a recent study found in the Northern province [49].

## Conclusion and Policy Implications

This study aimed to assess trends in childhood stunting inequalities in Rwanda between 2000 and 2019. While overall stunting prevalence has significantly declined between 2000 and 2019, the burden remains disproportionately high among children from poorer households, less-educated mothers, rural areas, boys, and all provinces except Kigali City. The study highlights that economic and educational disparities have exacerbated over time, with wealth inequalities showing the sharpest widening. These findings suggest that current pro-poor interventions may not efficiently address structural inequalities in childhood nutrition.

To bridge these gaps, targeted policies should focus on socioeconomic strengthening programs that reach the most vulnerable subgroups. Without urgent action, Rwanda risks missing its 2030 global nutrition target to reduce the prevalence of childhood stunting by 40%. Future studies should explore the contributors to these inequalities and evaluate the effectiveness of current intervention programs to inform more equitable and sustainable policy solutions.

## Supplementary Information

Below is the link to the electronic supplementary material.

Supplementary Material 1

## Data Availability

No datasets were generated or analysed during the current study.

## Abbreviations

CIX: Relative Concentration Index
LMICs: Low- and middle-income countries
PAF: Population Attributable Fraction
PAR: Population Attributable Risk
RDHS: Rwanda Demographic and Health Survey
SII: Slope index of inequality
VUP: Vision 2020 Umurenge Program
WMDM: Weighted Mean Difference from the Mean
