# Supplementary material for: Socio-Economic Inequalities of Childhood Stunting in Rwanda: Time Trend Analysis of Demographic and Health Surveys, 2000 to 2019
Source: J Epidemiol Glob Health. 2026 Apr 29;16(1):73. doi: 10.1007/s44197-026-00566-3 (PMC13275952; doi:10.1007/s44197-026-00566-3)
Supplement: Supplementary file 1 — Supplementary Material 1 [file 44197_2026_566_MOESM1_ESM.docx]

**Appendix 1. The trend in childhood stunting inequalities across different measures in Rwanda, 2000-2019**

| **Dimensions** | **Measures** | **2000** | **2005** | **2010** | **2014** | **2019** |
| --- | --- | --- | --- | --- | --- | --- |
| *Economic status* (Q5: ref.) | Difference | 19.6 | 25.7 | 28.2 | 27.7 | 37.8 |
|  | Ratio | 1.6 | 1.7 | 2.1 | 2.3 | 4.5 |
|  | CIX | -9.4 | -9.1 | -12.1 | -15.2 | -21.2 |
|  | SII | -21.7 | -25.1 | -30.9 | -33.2 | -41.8 |
|  | PAR | 14.9 | 16.1 | 18.9 | 16.8 | 23.2 |
|  | PAF | 32.1 | 31.9 | 43.0 | 44.3 | 69.0 |
| *Woman’s education* (secondary or higher: ref.) | Difference | 21.5 | 17.4 | 29.0 | 27.7 | 24.3 |
|  | Ratio | 1.7 | 1.5 | 2.2 | 2.4 | 2.2 |
|  | CIX | -4.4 | -3.6 | -5.5 | -7.7 | -9.2 |
|  | SII | -20.9 | -16.3 | -26.8 | -30.5 | -29.7 |
|  | PAR | 18.1 | 15.2 | 21.7 | 17.9 | 12.6 |
|  | PAF | 39.1 | 30.3 | 49.4 | 47.4 | 37.7 |
| *Place of residence* (Urban: ref.) | Difference | 17.3 | 15.1 | 19.4 | 16.7 | 16.0 |
|  | Ratio | 1.5 | 1.4 | 1.7 | 1.7 | 1.8 |
|  | PAR | 14.8 | 11.2 | 17.4 | 13.0 | 13.0 |
|  | PAF | 31.7 | 22.1 | 39.6 | 34.3 | 38.7 |
| *Child’s Sex* (Female: ref.) | Difference | 5.1 | 2.5 | 6.5 | 9.7 | 7.8 |
|  | Ratio | 1.1 | 1.1 | 1.2 | 1.3 | 1.3 |
|  | PAR | 2.5 | 1.3 | 3.4 | 4.8 | 3.6 |
|  | PAF | 5.4 | 2.6 | 7.8 | 12.6 | 10.9 |
| *Province* (Kigali City : ref.) | WMDM | 5.3 | 5.4 | 5.2 | 5.1 | 5.4 |
|  | PAR | 17.9 | 16.6 | 20.1 | 14.3 | 12.9 |
|  | PAF | 38.4 | 32.9 | 45.6 | 37.6 | 38.3 |

**Ref.**: reference category (with the lowest stunting prevalence). **CIX**: concentration index, **SII**: slope index of inequality, **PAR**: population attributable risk, **PAF**: population attributable fraction, WMDM: weighted mean difference from the mean.
